# Improving treatment decision-making in bipolar II disorder: a phase II randomised controlled trial of an online patient decision-aid

**DOI:** 10.1186/s12888-020-02845-0

**Published:** 2020-09-17

**Authors:** Alana Fisher, Rachael Keast, Daniel Costa, Louise Sharpe, Vijaya Manicavasagar, Josephine Anderson, Ilona Juraskova

**Affiliations:** 1 The University of Sydney, The School of Psychology, Sydney, NSW 2006 Australia; 2 The University of Sydney, The Matilda Centre for Research in Mental Health and Substance Use, Sydney, NSW 2006 Australia; 3 The University of Sydney, The Centre for Medical Psychology and Evidence-based Decision-making (CeMPED), Sydney, NSW 2006 Australia; 4 The Black Dog Institute, University of New South Wales, Sydney, NSW 2052 Australia

**Keywords:** Patient decision aid, Bipolar II disorder, Shared decision-making, Phase II randomised controlled trial, Decisional conflict, Patient knowledge, Patient involvement, Informed choice, Treatment, Relapse prevention

## Abstract

**Background:**

Many patients with bipolar II disorder (BPII) prefer to be more informed and involved in their treatment decision-making than they currently are. Limited knowledge and involvement in one’s treatment is also likely to compromise optimal BPII management. This Phase II RCT aimed to evaluate the acceptability, feasibility, and safety of a world-first patient decision-aid website (e-DA) to improve treatment decision-making regarding options for relapse prevention in BPII. The e-DA’s potential efficacy in terms of improving quality of the decision-making process and quality of the decision made was also explored.

**Methods:**

The e-DA was based on International Patient Decision-Aid Standards and developed via an iterative co-design process. Adults with BPII diagnosis (*n* = 352) were recruited through a specialist outpatient clinical service and the social media of leading mental health organisations. Participants were randomised (1:1) to receive standard information with/without the e-DA (Intervention versus Control). At baseline (T0), post-treatment decision (T1) and at 3 months’ post-decision follow-up (T2), participants completed a series of validated and purpose-designed questionnaires. Self-report and analytics data assessed the acceptability (e.g., perceived ease-of-use, usefulness; completed by Intervention participants only), safety (i.e., self-reported bipolar and/or anxiety symptoms), and feasibility of using the e-DA (% accessed). For all participants, questionnaires assessed constructs related to quality of the decision-making process (e.g., decisional conflict) and quality of the decision made (e.g., knowledge of treatment options and outcomes).

**Results:**

Intervention participants endorsed the e-DA as acceptable and feasible to use (82.1–94.6% item agreement); most self-reported using the e-DA either selectively (51.8%; relevant sections only) or thoroughly (34%). Exploratory analyses indicated the e-DA’s potential efficacy to improve decision-making quality; most between-group standardised mean differences (SMD) were small-to-moderate. The largest potential effects were detected for objective treatment knowledge (− 0.69, 95% CIs − 1.04, − 0.33 at T1; and − 0.57, 95% CIs − 0.99,-0.14 at T2), decisional regret at T2 (0.42, 95% CIs 0.01, 0.84), preparation for decision-making at T1 (− 0.44, 95% CIs − 0.81, − 0.07), and the Decisional Conflict Scale Uncertainty subscale (0.42, 95% CIs 0.08, 0.08) and Total (0.36, 95% CIs 0.30, 0.69) scores, with all SMDs favouring the Intervention over the Control conditions. Regarding safety, e-DA use was not associated with worse bipolar symptoms or anxiety.

**Conclusion:**

The e-DA appears to be acceptable, feasible, safe and potentially efficacious at improving patients’ decision-making about BPII treatment. Findings also support the future adoption of the e-DA into patient care for BPII to foster treatment decisions based on the best available evidence *and* patient preferences.

**Trial registration:**

Australian New Zealand Clinical Trials Registry ACTRN12617000840381 (prospectively registered 07/06/2017).

## Background

Bipolar II disorder (BPII) is a chronic and highly burdensome mental health disorder characterised by recurring depressive and hypomanic episodes, and is associated with decreased quality of life and functioning [1] as well as increased self-harm and suicidality [2]. BPII has a prevalence of up to 5% in community samples, making it more common than bipolar I (BPI) disorder (~ 2.5% prevalence) [3]. Long-term treatment in BPII is mainly ‘prophylactic’; it focusses on preventing and/or reducing the frequency and severity of mood relapses through a combination of pharmacological and adjunctive psychological interventions [4]. However, compared to BPI disorder, there is limited research to support the superior efficacy of one treatment option over others in BPII [5], with most published clinical guidelines extrapolating BPII treatment recommendations from the management of BPI disorder [6, 7]. Moreover, commonly-prescribed pharmacological options in BPII, including atypical antipsychotics (e.g., quetiapine) and mood-stabilisers (e.g., lithium and lamotrigine) carry a range of side effects [8] which may adversely impact on the person’s functioning and wellbeing [9], and thus need to be considered together with their respective benefits.

Shared decision-making (SDM) is increasingly recognised as the “gold standard” approach both in the medical and mental health settings [10, 11], and ensures that patient preferences for treatment are factored into cost-benefit evaluations of the viable treatment options available [12]. SDM involves the clinician, the patient, and often their family partnering together to make mutually agreed-upon treatment decisions that draw upon the clinician’s expertise and judgement as well as the patient’s (and their family’s) lived experience of the illness and treatment preferences. Patient decision-aids (DAs) are evidence-based interventions which can enhance SDM, as they provide patients with information and structured guidance to enable them to be more actively involved in making informed and values-based treatment decisions. A fast growing body of evidence demonstrates that DAs are acceptable, feasible, safe and efficacious at improving treatment decision-making in the inpatient, outpatient and primary care settings across a range of mental illnesses such as schizophrenia [13, 14] and unipolar depression [15, 16].

Our team was the first to develop a patient DA booklet for people with BPII [17], designed to improve SDM regarding relapse prevention in this population. The evidence-based DA was piloted, via a pre−/post-design, in a sample of 31 outpatients with BPII and 10 family members [17]. The pilot findings were positive, indicating that participants perceived the DA to be acceptable, useful and free from harm in making treatment decisions that were based on both adequate treatment knowledge and aligned with their treatment values. To facilitate the dissemination of the DA, the booklet was converted into an interactive website format (known as an online decision-aid or “e-DA”) (https://bipolardecisionaid.com.au/) [17].

In line with our published protocol paper [18], this study aimed to evaluate, via a Phase II randomised controlled trial (RCT), the e-DA’s acceptability, feasibility, and safety amongst people with BPII who are deciding on treatment options for relapse prevention. The potential efficacy of the e-DA at improving the quality of decision-making and quality of the decision made regarding BPII treatment was explored as a secondary study aim.

## Methods

### Study design

This pilot/feasibility Randomised Controlled Trial (RCT) was originally designed to determine the acceptability, feasibility, and safety of a world-first e-DA in an outpatient clinical setting. Due to the unanticipated restructure of the study site, revisions to the protocol (see procedure) were made and the trial was expanded to include an online setting. The inclusion of online participants considerably increased the trial sample size and thus permitted exploration of the potential efficacy of the e-DA. Simple randomisation occurred at the individual participant level and did not include blocking. As such, participants were parallel randomised (1:1) to receive access to either the e-DA (Intervention) or existing online information resources about BPII treatment (active Control).

Questionnaires were administered at baseline (T0), post-decision (T1) and 3-month follow-up (T2), with T1 as the primary assessment timepoint. The trial protocol, published elsewhere [18], was prospectively registered with the Australia and New Zealand Clinical Trials Registry (ACTRN12617000840381).

### Study setting and participants

#### Clinic-referred

Participants were recruited through the Black Dog Institute (BDI), a specialist outpatient mental health clinic in metropolitan Sydney, Australia (Pathway A). Eligible participants were 18+ years old with a confirmed clinical diagnosis of BPII whom were considering treatment options for maintaining mood-stability/preventing relapse. Exclusion criteria included lack of English proficiency, inability to provide informed consent to the study, were not experiencing an acute depressive or hypomanic episode, had no co-occurring alcohol/other drug use disorder or a neurological or major psychiatric condition, and who did not have computer/internet access. The exclusion of people with a co-occurring alcohol/other drug use disorder and/or psychiatric condition was because treatment considerations may differ for people with comorbid disorders as compared to those with a single disorder [19]. The BDI research-support staff referred interested patients to the University of Sydney (USYD) research team, who then registered them on the study website (https://bipolardecisionaid.com.au/).

#### Self-referred

Shortly after recruitment commenced, the BDI clinic underwent a significant restructure which resulted in unexpectedly slow recruitment rates. Therefore, participant recruitment was expanded to include an additional, self-referral recruitment pathway via study advertisements posted on social media sites, e-newsletters and websites of leading mental health organisations including the BDI, LIVIN’, and SANE Australia (Pathway B). Prospective self-referring participants then clicked on the link contained in the study advertisement which directed them to the study website and permitted them to self-register for the e-DA trial. For self-referring participants, it was not possible to have their BPII diagnosis confirmed by a clinician. In this way, we relied on self-reported BPII diagnosis. As was the case for clinic-referred participants, self-referring participants has to provide explicit information relating to their diagnosis including when they had been diagnosed by a clinician, the frequency of depressive and hypomanic episodes.

All participant data was collected online through the study website. Appropriate ethical approvals and site governance approvals were obtained to conduct the trial through the University of Sydney HREC (2016/763) and the Black Dog Institute Research Advisory Group (201,611 Fisher).

### Procedures

Participants were recruited for the trial between February 2018 and April 2019. In Pathway A, patients were introduced to the study after initial consultation with a psychiatrist at the BDI. Permission was obtained from interested eligible patients to share their contact details with the USYD research team. A USYD researcher (AF or RK) then contacted prospective participants to explain the study and confirm willingness to participate.

In Pathway B, participants self-registered to the study website by filling in a brief form which included name, email address and phone number. The participants then received an automated email which allowed them to activate their accounts and log in to the study website.

When they first logged in to the study website, participants from both pathways were directed to read the participant information statement and to complete the online written consent form. Consenting participants were directed to complete the baseline (T0) questionnaires. Upon completion of T0 measures, participants were randomly allocated (1:1) to either the Control or Intervention group, using a website-generated randomisation sequence, and had unlimited access to their allocated online website for the duration of the study.

Post-decision questionnaires were completed 3–4 weeks after T0 (T1); this timepoint coincided with when clinic participants were expected to have had their appointment with their managing community-based clinician to decide on current treatment options. A final set of questionnaires were completed 3 months post T1 follow-up (T2), at which time participants may have had a chance to review their treatment approach with their clinician. At each timepoint, participants were sent weekly site-generated email and text message reminders for up to 3 weeks after each set of questionnaires became available.

### Materials and measures

#### Intervention and control materials

**Fig. 1:**
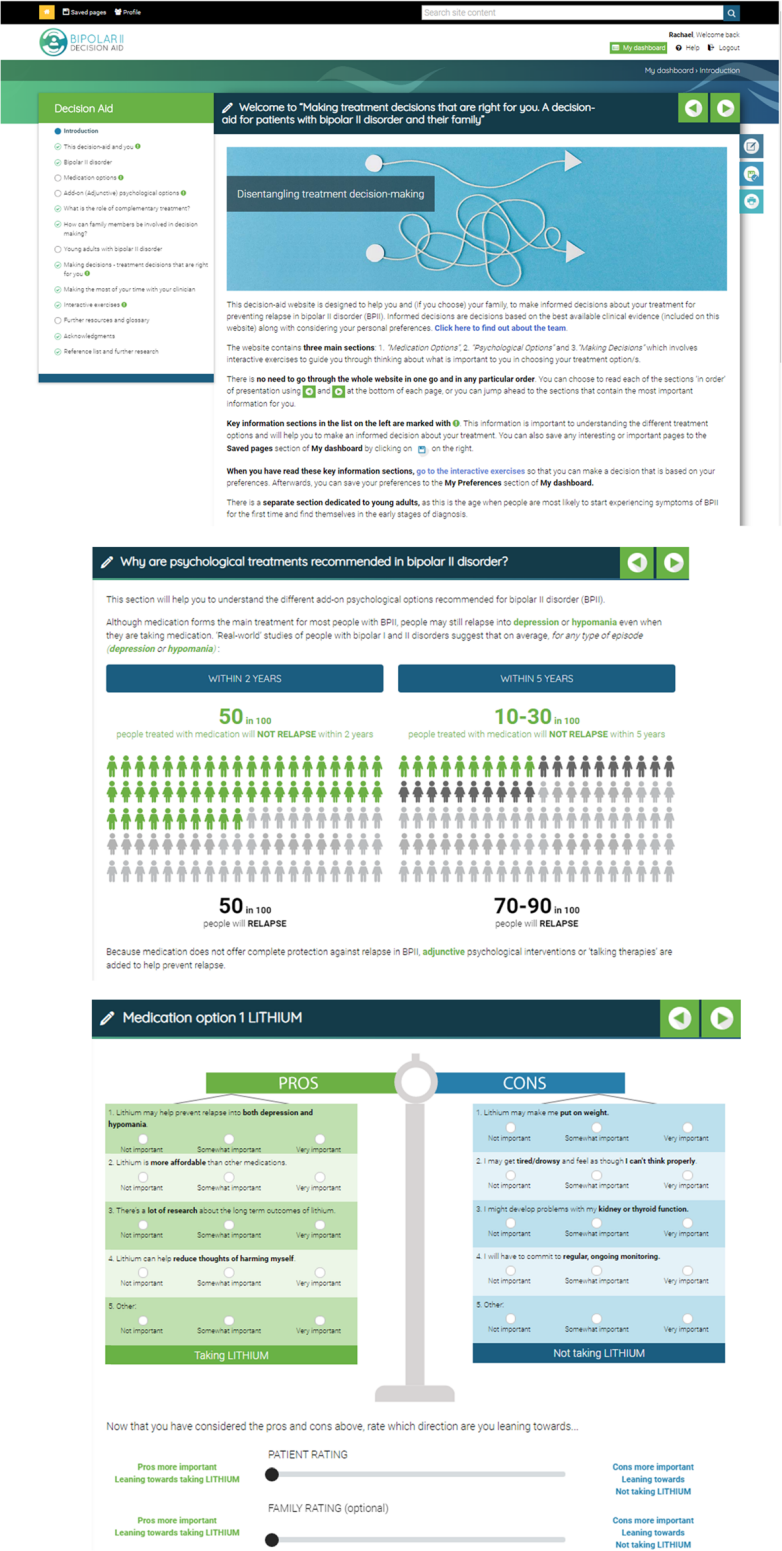
Screenshots of key pages from e-DA; introduction page, risk communication graphics, values clarification exercises

Participants in the Intervention group were given unlimited access to the e-DA (see Fig. 1). The development of this web-based resource has been described in detail elsewhere [17, 18]. In short, the e-DA involved a co-design process which consisted of qualitative decisional needs assessment interviews with key stakeholders (patients, their family members, and clinicians) [20–22], consultation and iterative review of prototypes with an expert working party of key stakeholders, further refinements and revisions based on structured interviews with newly-diagnosed young people, and functionality/usability testing and updates to the e-DA amongst key stakeholders [17, 18]. The e-DA provides evidence-based, unbiased and non-directional information on the first-line medication and adjunctive psychological treatment options for reducing relapse risk in BPII disorder. The e-DA uses lay language which was professionally copy-edited for low-health literacy levels; it includes a combination of text and graphics (e.g. risk communication diagrams, Fig. 1). Interactive values clarification exercises assisted participants to “weigh up” the relative importance of the positive (benefits) and negative features (side-effects, risks, other costs) of each of the presented treatment options, against their personal preferences and circumstances (see Fig. 1). A number of e-DA pages (e.g., introduction to available treatment options, summary of advantages and disadvantages of treatment options) were marked with (!) indicating that information presented on the page was considered important for making an informed treatment decision. Within the e-DA were practical prompts for collaborative decision-making approaches; these included a section on “Making the most of your time with your clinician” which presented the Ask-Share-Know questions to encourage more active patient involvement and better quality information exchange with clinicians [23, 24], as well as a section with instructions on how to involve a clinician and/or family member in the use of the values clarification exercises for shared deliberation of options.

Participants in the Control group were provided access to publicly available, evidence-based information on treatment options for bipolar disorder on the BDI website (https://www.blackdoginstitute.org.au/clinical-resources/bipolar-disorder/treatment). Both Intervention and Control participants were provided access to their allocated online materials via the same landing page, which was designed to increase the similarity between conditions. Both the e-DA and BDI website (active control) materials were designed for self-directed use by the patient, without guidance or coaching from a clinician. This said, the introduction to the e-DA stated that this resource was designed to supplement rather than replace discussions with a clinician.

#### Measures of feasibility, acceptability and safety

Participants’ perceptions of e-DA’s acceptability and utility was collected at T1, with 24 questions adapted from a previous e-mental health site evaluation [25]. Participants indicated their level of agreement on each item, which related to the e-DA’s perceived ease of use/usability, usefulness, information trustworthiness/balance versus bias, as well as attitudes towards use and likelihood of recommending to others.

Feasibility/ participant use of the e-DA was measured at T1 through a self-report question asking about participants’ level of use of the e-DA. Other usage data was also extracted from the e-DA’s inbuilt website analytics, i.e., participant’s level of completion of the values clarification exercises (< 1, 1, 2, 3, all 4 completed) and whether they visited the e-DA pages marked as “Key Information”. These measures only covered the participant’s own use of the e-DA, rather than if the participant had used the e-DA with a third party (e.g., their clinician and/or family member).

Safety measures included the Internal States Scale (ISS) measure of bipolar-related symptoms [26] and the short-form State Trait Anxiety Inventory (STAI) [27]. These measures were administered at both T1 and T2, to monitor whether e-DA use led to negative changes in bipolar symptom severity or state anxiety.

#### Measures of the quality of the decision-making process

Decisional conflict was measured at T1 with the 16-item Decisional Conflict Scale (DCS) [28]. The scale consisted of 5 subscales capturing participants’ feelings of being informed and certain about their options, as well as values clarity, support and ability to make and implement effective treatment decisions. The scale and subscales were each scored out of 100, with higher scores indicating greater difficulties in treatment decision-making.

Concordance between preferred and actual levels of decision-making involvement was determined by comparing participant responses to the single-item adapted Control Preferences Scale (CPS) [29, 30] at T0 (preferred level) and at T1 and T2 (actual levels) [31].

Preparedness for making treatment decisions at T1 was assessed using the 10-item Preparation for Decision-making Scale [32]. Higher scores (out of 100) represented higher perceived level of preparation for treatment decision-making.

(Subjective) Understanding of treatment options and outcomes was assessed at T1 and T2 with a purpose-designed 15 Likert-type item questionnaire covering the domains recommended by then current NHMRC Guidelines for Medical Practitioners on Providing Information to Patients (e.g., known effectiveness and common side-effects of options) [33]. Mean scores were calculated (possible range 1–5), with higher scores signifying better understanding of treatment options for BPII.

Regret or remorse associated with treatment decision was measured at T2, using the 5-item Decisional Regret Scale [34], scored out of 100. Higher scores indicated that participants had more remorse related to their treatment decision.

#### Measures of quality of the decision made

(Objective) Knowledge of treatment options and outcomes was measured at T1 and T2 knowledge questionnaire consisting of 14 forced-choice items covering both conceptual/gist-based knowledge and numerical/verbatim knowledge, again based on knowledge domains in the aforementioned NHMRC guidelines for informing patients [33].

Values-based, Informed choice was measured at T1 using a composite measure adapted from Marteau et al. [35], and based on Smith et al.’s theoretical framework for measuring knowledge in DA studies [36]. Participants were regarded as having made a values-based, informed-choice if they demonstrated adequate levels of objective knowledge^1^ of treatment options and outcomes, in conjunction with a treatment preference/choice that aligned with their self-reported positive or negative attitudes towards pharmacological and psychological treatments [35]. These attitudes were assessed by participants rating their level of agreement on eight Likert-type items, each anchored by a pair of opposing adjectives to describe medication or psychological treatment (e.g., medication is ‘important’/‘unimportant’) [35].

Uptake of effective treatment options was assessed at T1 with one question that asked participants to select which medication and/or psychological option/s they chose (if any).

#### Other measures

Baseline demographic and clinical information including age, education, time of BPII diagnosis, current treatments and current BPII symptoms was collected at T0 with a purpose-designed, self-report questionnaire.

### Sample size calculation

A rationale for the original sample size calculation (*n* = 20 per group; *N* = 92) is published elsewhere [18]. In light of changes to recruitment methods, and addition of a self-referral pathway online (see *Study setting and participants* and *Procedure*), the sample size was increased. The increased sample size was to account for expected lower retention rates at follow-up as a function study design/setting in DA trials in mental health (up to ~ 60–100% for clinic referrals [15, 37] versus 38% for self-referrals at post-DA use [25]). Assuming a conservative completion rate of 38% at T1, and the required minimum of 20 participants at T2, we calculated a sample size of *N* = 278 (i.e., *n* = 139 per group at T0, *n* = 52 at T1, *n* = 20 at T2). Due to higher than expected retention rates at T1 (≥ 50% see Results), we ceased recruitment once the recruited number of participants was reached.

### Statistical analyses

All analyses were conducted using SPSS version 26. Analysis focused the description of the acceptability and feasibility outcomes as well as assessing the e-DA’s potential efficacy at improving decision-making quality, comparing the e-DA Intervention group to the Control (usual care) group. Descriptive statistics, including means, standard deviations, and standardised mean differences examined all outcomes that were approximately continuous, whilst medians and inter-quartile ranges were used for ordinal variables. For nominal variables, frequencies and Odds Ratios (ORs) were calculated. The standardised mean difference was calculated as the difference between groups in mean change (from baseline, T0) at post-decision (T1) and after 3 months (T2), where applicable. Cohen’s guidelines [38] were used to interpret the magnitude of the standardised mean differences, where values around ±0.2 are considered small, ±0.5 moderate, and ± 0.8 large, and ORs, where a value of < 1.5 is considered a small effect or weak association, 1.5–5 moderate, and > 5.0 large/strong [39]. As this study was originally designed as a feasibility (Phase II) rather than efficacy (Phase III) trial, significance testing was not undertaken; however, 95% confidence intervals (CIs) were calculated for all between-group SMDs and ORs (see Tables 5, 6 and 7).

## Results

**Fig. 2:**
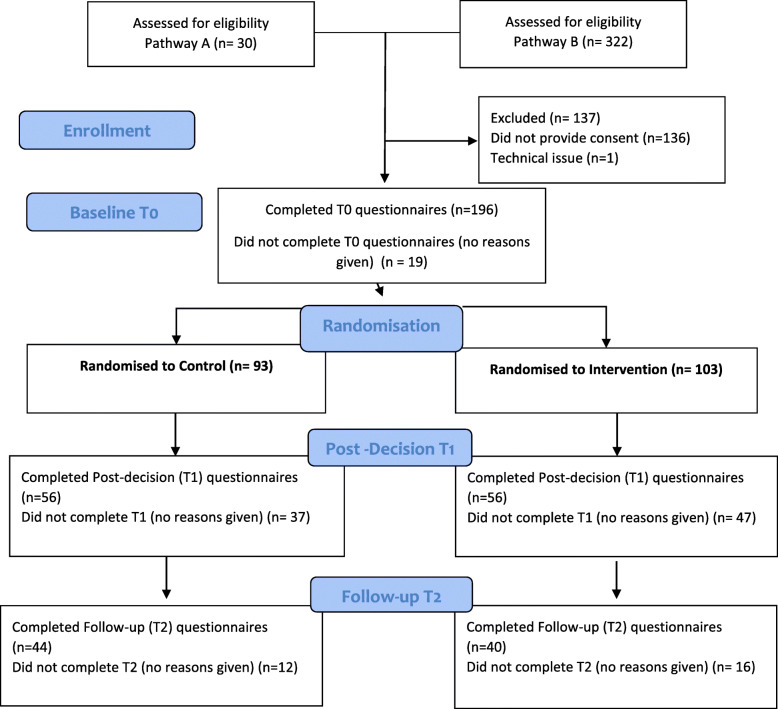
CONSORT Flow Diagram

Thirty patients expressed interest in participating in the study at the BDI clinic (Pathway A), and a further 322 participants self-registered through the study website (Pathway B). Of these 352 prospective participants, 136 declined to participate (i.e. did not provide consent) and one experienced a technical issue preventing access. Two hundred and fifteen participants were directed to complete baseline (T0) measures, of whom 196 completed these (91% completion rate). Participants were then randomised to the Intervention (*n* = 103) and Control (*n* = 93) groups. At T1, 56 participants completed measures in both the Intervention and Control groups (50 and 54% completion rates, respectively). At T2, 40 Intervention participants and 44 Control participants completed measures (36 and 42% completion rates, respectively). See CONSORT flow diagram (Fig. 2) for summary of participant flow. Given the online nature of the study and auto-generation of participant reminders, reasons for participant attrition could not be established. Where participants partially completed questionnaires at T1 and T2, their data was still included in the analyses where appropriate (e.g. completed one measure but did not complete subsequent measures). Accordingly, the n-values in tables do not always align with those noted in the CONSORT diagram.

The baseline sociodemographic characteristics of participants are summarised in Table 1. Control and Intervention participants were aged on average 39.28 (*SD* = 12.73) years and 39.40 (*SD* = 11.7) years, respectively. Most participants were female (74.2% Control, 70.9% Intervention), university-educated (54.9% Control, 60.5% Intervention), born in Australia (76.3% Control, 61.2% Intervention) and spoke English as their primary language at home (95.7% Control, 96.1% Intervention). Self-referral was the primary study pathway (93.5% Control, 92.2% Intervention).

**Table 1:** Demographic characteristics of participants in the Control (*n* = 93) and Intervention (*n* = 103) groups at baseline (T0)

	Control	Intervention
	***M (SD)***	***M (SD)***
**Age**	39.38 (12.73)	39.40 (11.06)
	***n (%)***	***n (%)***
**Gender**		
Female	69 (74.2)	73 (70.9)
Male	24 (25.8)	30 (29.1)
**Highest qualification**		
Year 12 / HSC or below	13 (14.0)	18 (17.5)
TAFE certificate / diploma	29 (31.2)	33 (32.0)
University degree	33 (35.5)	34 (33.0)
Postgraduate degree	18 (19.4)	18 (17.5)
**Current employment**		
Working full-time	33 (35.5)	34 (33.0)
Working part-time	13 (14.0)	21 (20.4)
On sick leave	17 (18.3)	17 (16.5)
Not employed/retired/home duties	12 (12.9)	17 (16.5)
Studying	13 (14.0)	9 (8.7)
Other (e.g., casual employment, combination)	5 (5.4)	5 (4.9)
**Country of birth**		
Australia	71 (76.3)	63 (61.2)
Other (e.g., New Zealand, USA, UK, Canada)	22 (23.7)	40 (38.8)
**Language spoken at home**		
English	89 (95.7)	99 (96.1)
Other (e.g., Cantonese, Portuguese)	4 (4.3)	4 (3.9)
**Current relationship status**		
Single/dating	39 (41.9)	32 (31.1)
Married/living with partner	43 (46.2)	60 (58.3)
Separated or divorced	9 (9.7)	10 (9.7)
Widowed	2 (2.2)	1 (1.0)
**Marital status changed since BPII diagnosis** (yes)	18 (19.4)	27 (26.2)
**Children** (yes)	40 (43.0)	58 (56.3)
**Current living arrangements**		
By yourself/independently	20 (21.5)	19 (18.4)
With partner (with/out children)	46 (49.5)	59 (57.3)
With children only	2 (2.2)	7 (6.8)
With other family member/s	13 (14.0)	10 (9.7)
With non-family members	10 (10.8)	5 (4.9)
Other (e.g., Combination of above)	2 (2.2)	3 (2.9)
**Referral pathway**		
Self-referral online	87 (93.5)	95 (92.2)
Clinic referral	6 (6.5)	8 (7.8)

Almost half of participants (47.3% Control, 48.5% Intervention) had a longstanding diagnosis of BPII of more than 5 years, whilst a small proportion of the sample had received their BPII diagnosis within the past year (15.1% Control, 19.4% Intervention; See Table 2). Most participants reported being diagnosed with another psychiatric disorder prior to their BPII diagnosis (e.g., unipolar depression and/or anxiety disorder), which was later superseded by their BPII diagnosis (71% Control, 67% Intervention). Participants most commonly saw a psychiatrist or a GP regarding their BPII (92.5, 82.8% Control, 97.1, 85.4% Intervention), and usually attended consultations alone (84.9% Control, 80.6% Intervention) with family most commonly attending at least one consultation (52.7% Control, 46.6%).

**Table 2:** Clinical characteristics of participants in the Control (*n* = 93) and Intervention (*n* = 103) groups at baseline (T0)

	Control	Intervention
	***M*** (***SD***)	***M*** (***SD***)
**Age diagnosed with BPII**	32.10 (12.29)	32.54 (10.05)
	**n (%)**	**n (%)**
**Years since diagnosis**		
< 1 year	14 (15.1)	20 (19.4)
1–5 years	35 (37.6)	33 (32.0)
> 5 years	44 (47.3)	50 (48.5)
**Previous mental health diagnosis other than BPII** (yes)	66 (71.0)	69 (67.0)
**BPII episodes (perceived) frequency**		
Rapid cycling (4 or more times per year)	58 (62.4)	66 (64.1)
2–3 times per year	25 (26.9)	25 (24.3)
Once per year or less often	10 (10.7)	12 (11.7)
**BPII episodes (perceived) type**		
Mainly depressive episodes	48 (51.6)	49 (47.6)
Equal depression/hypomania	35 (37.6)	41 (39.8)
Mainly hypomanic episodes	4 (4.3)	8 (7.8)
Mainly euthymic/subsyndromal	6 (6.5)	5 (4.9)
**Current medication** (Yes; e.g., Lithium, Lamotrigine)	76 (81.7)	83 (80.6)
**Current psychological treatment (**Yes; e.g., CBT, ACT)	53 (57.0)	58 (56.3)
**Current goal of BPII treatment**		
Prevent recurrence/relapse	69 (74.2)	78 (75.7)
Treat current depression/hypomania	15 (16.2)	18 (17.5)
Other (e.g., Reduce length and severity of episodes)	9 (9.7)	7 (6.8)
**Other chronic medical conditions** (Yes)	40 (43.0)	37 (35.9)
**Health professionals seen about BPII (> 1 may apply)**		
Psychiatrist	86 (92.5)	100 (97.1)
GP	77 (82.8)	88 (85.4)
Psychologist	69 (74.2)	70 (68.0)
Counsellor	22 (23.7)	31 (30.1)
Mental health care nurse	22 (23.7)	24 (23.3)
Other health professional (e.g., mental health support worker, peer support worker, acupuncturist)	4 (4.3)	6 (5.8)
**Family attended consultation/s** (Yes)	49 (52.7)	48 (46.6)
**Usual attendance in consultations**		
Patient alone	79 (84.9)	83 (80.6)
Sometimes alone or accompanied	12 (12.9)	14 (13.6)
Attends accompanied	2 (2.2)	6 (5.8)

Participants in both groups had strong information preferences and most wanted “as much information as possible, regardless of whether it was good or bad news” (90.3% Control, 93.2% Intervention). Many participants were at the stage of their decision-making where they were either actively considering (36.6% Control, 42.7% Intervention) or open to reconsidering their treatment options (48.4% Control, 40.8% Intervention). Most participants reported no health literacy-related difficulties (89.2% Control, 89.3% Intervention) (Table 3).

**Table 3:** Decision-making-related characteristics in the Control (*n* = 93) and Intervention (*n* = 103) groups at T0

	Control	Intervention
	***M (SD)***	***M (SD)***
**Information preferences –** ***amount*** **(/5)**	4.83 (0.52)	4.76 (0.57)
	***n (%)***	***n (%)***
**Information preferences –** ***type***		
As much information as possible, good and bad	84 (90.3)	96 (93.2)
Only information needed to care for myself properly	6 (6.5)	6 (5.8)
Additional information only if it is good news	3 (3.2)	1 (1.0)
**Stage of treatment decision making**		
Not begun to think about the choices for treatment	0 (0.0)	4 (3.9)
Not begun to think about treatment choices but interested	3 (3.2)	2 (1.9)
Considering/reconsidering treatment options	79 (85.0)	86 (83.5)
Already made a treatment decision, unlikely to change mind	11 (11.8)	11 (10.7)
**Health literacy difficulties**		
None	83 (89.2)	92 (89.3)
Some	10 (10.8)	11 (10.7)
**Preferred involvement in decision-making (triadic)**		
Patient-led with/out clinician/family	57 (61.3)	55 (54.4)
Shared/collaborative with clinician/family	18 (19.4)	24 (23.3)
Family-led with patient and clinician	2 (2.2)	2 (1.9)
Clinician-led with/without patient/family	16 (17.2)	21 (20.4)

Across all four acceptability domains, almost all Intervention participants indicated positive feedback on using the e-DA, with high levels of agreement on: perceived ease of use of the e-DA (82.1–94.6%), perceived usefulness (71.4–92.9%), positive attitudes towards use (83.9–91.1%), and perceived trustworthiness of the information in the e-DA (89.3–92.9%) (see Table 4). Regarding feasibility, 85.7% of Intervention participants self-reported use of the e-DA, and reasons for non-use related to technical/internet access issues and symptoms (e.g., difficulties with memory and motivation). Site analytic data revealed that 38.8% of Intervention participants consulted at least 50% of all the e-DA pages containing important information (i.e., pages marked with an exclamation mark [!] icon), whilst 23.3% of participants completed at least one of the values clarification exercises with a small minority (4.9%) completing these worksheets for all four treatment options.

**Table 4:** Acceptability, feasibility and perceived utility of the e-decision-aid’s (DA) in the Intervention group (*n* = 56, T1)

	Agree/Somewhat Agree	Disagree/Somewhat disagree
	**n (%)**	**n (%)**
**Perceived ease of use of e-DA**		
Font easy-to-use	50 (89.3)	6 (10.7)
Easy-to-use	50 (89.3)	6 (10.7)
Clearly organised information	51 (91.1)	5 (8.9)
Design appealing	51 (91.1)	5 (8.9)
Easy-to-understand information	52 (92.9)	4 (7.1)
Colours pleasant	53 (94.6)	3 (5.4)
Pictures pleasant	52 (92.9)	4 (7.1)
Important information easy-to-find	46 (82.1)	10 (17.9)
**Perceived usefulness of e-DA**		
Content interesting	52 (92.9)	4 (7.1)
Useful in making a treatment decision	50 (89.3)	6 (10.7)
Right amount of information included	47 (83.9)	9 (16.1)
Information I needed included	49 (87.5)	7 (12.5)
Helped with my concerns	46 (82.1)	10 (17.9)
Found links to information and other resources	47 (83.9)	9 (16.1)
Learnt something new	46 (82.1)	10 (17.9)
Made it easier to discuss treatment options with (my) clinician	48 (85.7)	8 (14.3)
Made it easier to discuss treatment options with family	40 (71.4)	16 (28.6)
**Attitudes towards using e-DA**		
Would recommend to others in my situation	51 (91.1)	5 (8.9)
Would go back and re-read sections	51 (91.1)	5 (8.9)
Information did *not* make me anxious	47 (83.9)	9 (16.1)
**Perceived trustworthiness and balance of information in e-DA**		
Information trustworthy	52 (92.9)	4 (7.1)
Information up-to-date	52 (92.9)	4 (7.1)
Equal emphasis on each of the medication options	52 (92.9)	4 (7.1)
Equal emphasis on each of the adjunctive psychological options	50 (89.3)	6 (10.7)
	**n (%)**	
**Access of the e-DA** (self-report)		
Yes, all pages/sections	10 (17.9)	–
Yes, quite thoroughly	9 (16.1)	–
Yes, briefly/only sections I felt relevant to me	29 (51.8)	–
No, because… (e.g., forgot to, technical/internet problems)	9 (16.1)	–
**Visits to key information pages on site** (yes)		
All pages visited	16 (15.5%)	–
>/= 50% pages visited	24 (23.3%)	–
< 50% pages visited	63 (61.2%)	–
**Use of Values Clarification Exercises**		
< 1 treatment option exercise completed	79 (76.7%)	–
1–3 treatment option exercise/s completed	19 (18.4%)	–
All 4 treatment option exercises completed	5 (4.9%)	–

Regarding safety of e-DA use, the standardised mean differences (SMD) between the Intervention and Control groups on state anxiety and all bipolar symptom severity subscale measures were small in magnitude, at both T1 or T2 (*SMD* range = -0.20 – 0.37; see Table 5). Further, all between group differences (except for the ‘Activation’ Bipolar symptom subscale score at T2), were in favour of the Intervention group.

**Table 5:** Safety measures at post treatment decision (T1) and at 3-month follow-up (T2)

	Post-treatment decision (T1)			3-month follow-up (T2)		
	Control	Intervention	***SMD*** (95% CI)	Control	Intervention	***SMD*** (95% CI)
	***M (SD, N)***	***M (SD, N)***		***M (SD, N)***	***M (SD, N)***	
**Internal State Scale**						
Perceived Conflict (/500)	185 (102.44, 56)	162.98 (115.68, 57)	0.20 (−0.17, 0.57)	166.59 (101.04, 44)	155.26 (112.92, 38)	0.11 (−0.33, 0.55)
Well Being (/300)	95.18 (47.33, 56)	102.46 (48.23, 57)	−0.15 (− 0.52, 0.22)	119.09 (73.1, 44)	134.21 (75.5, 38)	− 0.20 (− 0.64, 0.24)
Activation (/500)	185.36 (105.76, 56)	177.72 (140.98, 57)	0.06 (−0.31, 0.43)	161.59 (108.5, 44)	186.58 (141.01, 38)	−0.20 (− 0.64, 0.24)
Depression (/200)	90.71 (60.45, 56)	82.81 (63.74, 57)	0.13 (−0.25, 0.50)	88.86 (63.91, 44)	69.21 (58.79, 38)	0.32 (−0.12, 0.76)
**State-Trait Anxiety Inventory (20–80)**	50.67 (14.45, 55)	47.08 (14.99, 57)	0.24 (−0.13, 0.62)	51.21 (15.65, 44)	45.44 (15.12, 38)	0.37 (−0.07, 0.82)

*SMD* standardised mean difference

At T1, exploratory analyses revealed that Intervention participants reported lower total decisional conflict (M = 23.80/100, *SD* = 15.19) than Control participants (M = 30.19/100, *SD* = 20.1) (*SMD* = 0.36), with Intervention participants scoring lower on all five decisional conflict subscales (See Table 6). Whilst the magnitude of the between-group SMDs was small across all subscales, larger differences between groups were recorded on the on the Uncertainty subscale (*SMD =* 0.42), followed by the Uninformed (*SMD* = 0.31) and Unsupported subscales (*SMD* = 0.32) (Table 6). Intervention participants reported higher preparation for treatment decision-making (M = 62.08/100, *SD* = 24.31) than Control participants (M = 50.47/100, *SD* = 28.46) at T1 (*SMD* = -0.44). They also indicated less regret about their treatment decision at 3-month follow-up (Intervention M = 17.05, *SD* = 14.68; Control M = 25.11, *SD* = 22.95; *SMD* = 0.42). At both T1 and T2, there were only small between-group differences on participants’ subjective understanding of treatment options (*SMD* = -0.22 and − 0.10), and whether participants achieved their preferred level of involvement in decision-making about treatment with their clinician and family (*ORs* = 0.96 and 1.07).

**Table 6:** Quality of the decision-making process outcomes in the Control and Intervention groups post-treatment decision (T1) and at 3-month follow-up (T2)

	Post-treatment decision (T1)			3-month follow-up (T2)		
	Control	Intervention		Control	Intervention	
	***M*** **(*SD,*** **n)**	***M*** **(*SD,*** **n)**	***SMD*** **(95% CI)**	***M*** **(*SD,*** **n)**	***M*** **(*SD,*** **n)**	***SMD*** **(95% CI)**
**Decisional Conflict Scale (/100)**						
Total	30.19 (20.1, 69)	23.80 (15.19, 74)	0.36 (0.30, 0.69)	–	–	–
Uncertainty	41.91 (26.62, 69)	32.09 (20.26, 74)	0.42 (0.08, 0.08)	–	–	–
Uninformed	28.74 (25.31, 69)	21.73 (19.84, 74)	0.31 (−0.02, 0.64)	–	–	–
Unclear values	22.95 (20.88, 69)	18.81 (16.78, 74)	0.22 (−0.11, 0.55)	–	–	–
Unsupported	28.99 (22.31, 69)	22.75 (16.22, 74)	0.32 (−0.01, 0.65)	–	–	–
Effective decision	28.8 (21.89, 69)	23.65 (17.56, 74)	0.26 (−0.07, 0.59)	–	–	–
**Subjective understanding of treatment (/5)**	3.66 (0.84, 59)	3.84 (0.75, 66)	−0.22 (− 0.57, 0.14)	3.85 (0.67, 45)	3.93 (0.9, 38)	−0.10 (− 0.54, 0.33)
**Preparation for Decision-making Scale (/100)**	50.47 (28.46, 58)	62.08 (24.31, 59)	−0.44 (− 0.81, − 0.07)	–	–	–
**Decisional Regret Scale (/100)**	–	–	–	25.11 (22.95, 47)	17.05 (14.68, 44)	0.42 (0.01, 0.84)
	***‘yes’*** **n (%)**	***‘yes’*** **n (%)**	***OR*** **(95% CI)**	***‘yes’*** **n (%)**	***‘yes’*** **n (%)**	***OR*** **(95% CI)**
**Experienced preferred level of involvement in decisions (triadic)**	28 (39%)	30 (38%)	0.96 (0.50,1.86)	15 (32%)	12 (33%)	1.07 (0.45, 2.55)

Empty cells (−) denote outcome was not measured at that time point

*SMD* standardised mean difference, *OR* odds ration, *CI* confidence interval

At both T1 and at T2 follow-up, exploratory analyses revealed that moderate between-group differences on objective knowledge of treatment options and outcomes, with the Intervention group scoring higher that on measures of conceptual and numerical knowledge, and overall objective knowledge (*SMD* Range = − 0.52 to − 0.69; see Table 7). Regarding informed choice, a higher proportion of the Intervention than Control participants made an informed choice about medication/s (55% versus 39%, respectively; *OR* = 1.94) and adjunctive psychological options (59% versus 53%; *OR* = 1.31) that was both based on adequate knowledge and aligned with their treatment values (see Table 7). At T1, the majority of participants in both groups indicated uptake of an effective treatment option, including first-line medication with/without adjunctive psychological therapy (61% Intervention, 68% Control; OR = 0.75 see Table 7).

**Table 7:** Decision quality outcomes in the Control and Intervention groups post treatment decision (T1) and at 3-month follow-up (T2)

	Post-treatment decision (T1)			3-month follow-up (T2)		
	Control	Intervention		Control	Intervention	
	***M*** **(*SD*, n)**	***M*** **(*SD*, n)**	***SMD*** **(95% CI)**	***M*** **(*SD*, n)**	***M*** **(*SD*, n)**	***SMD*** **(95% CI)**
**Objective knowledge of treatment**						
Conceptual (/18)	10.51 (3.47, 67)	12.51 (4.01, 74)	−0.53 (−0.87, − 0.19)	11.91 (3.2, 47)	13.64 (3, 44)	−0.55 (− 0.98, − 0.13)
Numerical (/20)	13.31 (3.29, 62)	14.97 (3.17, 68)	− 0.52 (− 0.87, − 0.16)	12.83 (3.78, 47)	14.98 (2.76, 42)	−0.64 (−1.07, − 0.21)
Total (/38)	24.05 (5.33, 62)	27.79 (5.58, 68)	−0.69 (− 1.04, − 0.33)	24.74 (5.7, 47)	27.93 (5.53, 44)	−0.57 (− 0.99, − 0.14)
**Attitudes towards treatment**						
Medication (/28)	20.18 (5.73, 56)	20.3 (4.72, 57)	−0.02 (− 0.39, 0.35)	–	–	–
Psychological treatment (/28)	22.27 (4.63, 56)	21.32 (4.85, 57)	0.20 (− 0.17, 0.57)	–	–	–
	***‘yes’*** **n (%)**	***‘yes’*** **n (%)**	***OR*** **(95% CI)**	***‘yes’*** **n (%)**	***‘yes’*** **n (%)**	***OR*** **(95% CI)**
**Adequate treatment knowledge**	51 (82.3%)	64 (91.4%)	2.30 (0.80, 6.64)	41 (87.2%)	41 (97.6%)	6.00 (0.69, 52.07)
**Informed choice**						
Medication	17 (39%)	22 (55%)	1.94 (0.81, 4.63)	–	–	–
Psychological treatment	23 (53%)	23 (59%)	1.31 (0.55, 3.13)	–	–	–
**Uptake of effective treatment options** (i.e., first-line medication/s +/− adjunctive psychological therapies)	48 (67.6)	47 (61.0)	0.75 (0.38, 1.48)	–	–	–

“Adequate treatment knowledge” is defined as > 50% correct on objective knowledge of treatment items (i.e., at least 20 out of 38 marks). To have adequate knowledge, participants must either get correct: i) all conceptual knowledge items (18 marks) *plus* at least 2 marks on numerical knowledge items (2 marks); or ii) all numerical knowledge items (20 marks)

Empty cells (−) denote outcome was not measured at that time point

*SMD* standardised mean difference, *OR* odds ration, *CI* confidence interval

## Discussion

A large body of literature has already established the efficacy of DAs across a wide range of health conditions [40], and yet the use of DAs in clinical practice remains relative limited [41]. Therefore, the primary focus of the current trial was on establishing the e-DA’s acceptability, feasibility and safety, which are core requirements for the implementation of any intervention in clinical practice. This pilot trial was the first to establish that a patient decision-aid website (e-DA) was feasible, acceptable, and safe for patients with BII who are deciding on treatment options to reduce their risk of relapse. In addition, exploratory analyses revealed that e-DA’s potential efficacy to improve outcomes related to decision-making quality.

Developing an evidence-based intervention that is safe and can be feasibly adopted in clinical practice to support patients with BPII is a critical first step in bridging the persistent shared decision-making (SDM) gap between medical and psychiatric illnesses. It should be acknowledged that although patient DAs are designed to facilitate SDM, the provision of patient DAs in and of themselves does not guarantee that SDM occurs. Rather, SDM is complex and expansive process, especially in mental health [42, 43]. Therefore patient DAs should be regarded as a tool as part of suite of multicomponent interventions that can be used to encourage SDM [43]. It cannot be ascertained from the current trial whether the patient decision-aid was efficacious at improving SDM per se, however, it did lead to improved outcomes in both decision-making quality (e.g., decisional conflict) and quality of the decision made (e.g., treatment knowledge). Principal findings are discussed below in terms of the broader existing literature and their implications for helping to promote core elements of SDM in mental healthcare.

Of primary interest, the e-DA was shown to be feasibility and acceptable amongst potential end-users, and importantly was not associated with adverse effects on patient symptoms or anxiety (i.e., safety). The vast majority of patients found the e-DA easy to use, useful in making informed and collaborative treatment decisions, provided trustworthy and balanced information, and would recommend the resource to others in their situation. As was the case here, patients also strongly endorsed the content and format of the e-DA’s earlier iteration as a patient DA booklet [44], which suggests that a number of delivery modalities are acceptable to these patients and may be used interchangeably based on a patient’s circumstances and preferences at the time of decision-making (e.g., limited internet access, desire for anonymity). Further, a large majority of patients reported using the e-DA and over a third reported using it thoroughly and/or consulting all pages/sections. This self-report data was confirmed by site analytic user behaviour data, which showed over a third of patients consulted a majority of the webpages marked as important reading. Patient use of the values clarification exercises was lower, with only a quarter of patients completing at least one exercise to consider their options for treatment. Although values clarification exercises constitute a key component of patient decision-aids, little is known about patient usage of these exercises and which design features may enhance their usage [45, 46]. Finally, higher than expected completion rates (50 and 54% of Intervention and Control Groups at T1) lends further support to the feasibility and acceptability of this e-DA amongst potential end users. Current findings are therefore informative and provide integral support for the e-DA’s future implementation into clinical practice, including the potential need for additional guidance on using the values clarification exercises. This is important since implementation of patient DAs into clinical practice remains limited, with over half (55%) having no uptake post-RCT evaluation [41]. Since completion of this trial, the e-DA has been integrated into the Black Dog Institute website to ensure its ongoing maintenance and far-reaching dissemination to end-users.

Although the current study was initially designed as a feasibility pilot trial, unavoidable modifications to the study protocol permitted us to recruit more broadly, and with this the opportunity to conduct exploratory analyses of the e-DA’s potential efficacy on outcomes relevant to improved decision-making quality. Findings related to e-DA’s potential efficacy should be treated as preliminary however, given that the RCT was not designed nor powered to test efficacy. Moreover, the confidence intervals associated with most of the aforementioned effects were wide, indicating that these effects preclude any precise conclusions regarding the e-DA’s efficacy and should be interpreted with some caution.

This said, findings suggest the e-DA may be efficacious at reducing patients’ overall decisional conflict and increasing their objective knowledge of the available treatment options and outcomes for relapse prevention in BPII. Indeed, the positive effects of patient DAs on decisional conflict and treatment knowledge are amongst the effects with strongest evidence to support them, both in the mental health and in the medical DA literature [47]. Importantly too, observed increases in patient treatment knowledge appear to be maintained at 3-month follow-up. These are important outcomes insofar as poor understanding of bipolar illness and treatments is associated with treatment non-adherence [48, 49], which is in turn associated with a more recurrent illness [50].

Of note, only patients with access to the e-DA indicated low levels of decisional conflict consistent with implementing rather than delaying their treatment decision (*M* < 25). Importantly, patients without decisional conflict are also more likely to receive their preferred mental health treatment and be more satisfied with their care [51]. Of the decisional conflict subscales, the e-DA appeared to have moderate effects on patient’s indecision related to uncertainty about the treatment options. This suggests that e-DA may be helpful in clarifying which of the available options is “the best choice” for patients, as well increasing their confidence in making this choice. This may be of particular relevance to the BPII treatment setting, with there is no “single best choice” but rather a number of viable evidence-based options with different benefit-cost profiles [52]. Previous studies have demonstrated that the use of SDM can help patients to cope better with uncertainty regarding healthcare options [53], while increases in patient knowledge is a key modifiable factor in making inherently difficult decisions easier [54]. However in this study, in contrast to other DA literature [47], the e-DA only appeared to produce small effects on patients’ subjective understanding of treatment, and perceptions of being informed (on the DCS). One interpretation of these findings is that patients accessing publicly available information (via the clinic website) may overestimate their treatment knowledge, that is they feel well informed but less knowledgeable about treatment. It is imperative therefore that clinicians ask questions to ascertain that patient do have adequate knowledge when deciding on their treatment, or that patients are given opportunities to self-assess their knowledge to highlight any gaps.

The e-DA also appeared to have moderate effects on patients’ preparedness to make a treatment decision and regret about their actual treatment decision. Again these findings are consistent with the broader DA RCT literature [47] and may in fact be related, insofar as patients who feel well prepared to make decisions may have increased self-efficacy and satisfaction in the decision-making process [55], and thus feel less regretful about the outcome. Better preparedness to make treatment decisions aligns well with the fact that the e-DA was designed to be a self-guided rather than clinician-guided resource, for use in preparation for and/or between consultations with a clinician involving decision-making about treatment.

It is worth noting that this e-DA may lead to more limited improvements on some aspects of treatment decision-making, such as making an informed choice to take medication, and having adequate levels of knowledge regarding treatment options and outcomes. Given the chronicity of the current BPII sample, especially those patients that we self-referring (large proportion diagnosed 5+ years ago), it is possible that these patients are already equipped with the necessary skills and knowledge to make informed treatment choices. This is supported by the fact that this sample was highly information-seeking. Similarly, qualitative studies in BPII [20, 21] indicate that over time patients develop expertise in their illness and with this, are more likely to assume an active and informed decision-making role. Thus, the current e-DA may be especially useful to patients with a recent diagnosis, who are relatively naïve to the available treatment options and deciding on options for the first time. It is likely that a future RCT in a larger sample of newly diagnosed patients would yield findings that are consistent with other recent trials of patient DAs in mental health (e.g., [15, 37]). In light of the costly and resource-heavy nature of large-scale efficacy trials, more fruitful avenues for future research include testing whether the e-DA facilitates SDM within subsequent clinical consultations (e.g., via triangulation of patient-report, clinician-report, and observer-rating measures [56, 57], and testing implementation strategies to determine how the e-DA can be optimally embedded within clinical practice.

This study has a number of strengths, including a randomised-design with follow-up assessment, and a combination of self-report and objective assessments. There are, however, a number of limitations that also warrant consideration. Firstly, this mostly self-referring patient sample was largely highly-educated with strong preferences for information and without health literacy-related issues. Without measuring participants’ baseline knowledge at T0 we cannot account for the contribution of prior knowledge to scores at T1 and T2. On balance, it was decided that including too many baseline measures would be burdensome and may act as a barrier to participants accessing the e-DA. This said, moderate between group differences between the Intervention and Control groups at T1 suggests that the e-DA contributed to improvements in participants’ treatment knowledge, irrespective of their prior knowledge levels. The review of the e-DA for low health-literacy levels also increases our confidence that the information in the e-DA is broadly understandable and usable to people.

Further, given that majority of participants were self-referred rather than clinic-referred, we were unable to verify the accuracy of all patients’ BPII diagnosis and had to rely on self-report. Online self-referral methods are not unusual when assessing patient decision-support needs and/or evaluating online decision-support tools [25, 58, 59], and participant characteristics in the current sample mirror those of clinic-recruited participants with bipolar disorder [20, 60, 61]. Even though the opportunistic use of a multimodal recruitment strategy within this RCT increased sample heterogeneity, and fact the e-DA was copy-edited for low health literacy levels, the generalisability of current findings are unknown [62]. Moreover, the wide confidence intervals on some of the efficacy-related outcomes indicate that knowledge about the ‘true’ nature of the e-DA’s efficacy at improving decision-making is still limited. Since our study comprised a chronic patient sample with longstanding BPII diagnoses, the feasibility and acceptability of the BPII e-DA may be different in a patient sample with less chronic illness, who are deciding on treatment options for the first time. This said, qualitative research has elucidated the iterative, ongoing nature of treatment decision-making in BPII as a recurrent and episode illness [20], and indeed 85% of the sample reported that they were actively considering or reconsidering their treatment options. Finally, patient involvement in decision-making, as measured by the Control Preferences Scale, is unlikely to adequately capture whether or not SDM actually took place. SDM is a multidimensional construct and thus future research would benefit from the inclusion of a more comprehensive measure. Patient decision-making involvement is a key aspect of decision-making quality and patient decision-aid effectiveness [63], and was deemed sufficient in light of the fact that the current trial was focussed on feasibility.

## Conclusions

Compared to the physical illness domain, such as oncology, mental healthcare has been relatively slow in its promotion and adoption of shared decision-making (SDM) [10, 42, 64], with a paucity of rigorously evaluated decision-support tools such as patient DAs [47]. This is the first known study internationally to demonstrate the feasibility, acceptability, and safety of an online patient DA (e-DA) to support treatment decision-making about relapse prevention options in BPII. This study also yielded preliminary insights into the e-DA’s potential efficacy. Patients accessing the e-DA appeared more comfortable making a treatment decision (reporting less decisional conflict), more knowledgeable about the available treatment options and outcomes, better prepared to make a treatment decision and less regretful about said decision. The current e-DA is one of relatively few RCT-evaluated DAs for patients with mental health conditions [11, 47], and has the potential to assist patients to make informed treatment choices that are consistent with the best available evidence as well as their preferences for treatment.

## Data Availability

The datasets generated and/or analysed during the current study are not publicly available due to not having ethics approval, but are available from the corresponding author on reasonable request.

## Abbreviations

BPII: Bipolar II Disorder
CPS: Control Preferences Scale
DA: Patient Decision Aid
DCS: Decisional Conflict Scale
e-DA: Online Patient Decision Aid
ISS: Internal States Scale
OR: Odds Ratio
RCT: Randomised Controlled Trial
SDM: Shared Decision Making
SMD: Standardised Mean Difference
STAI: State Trait Anxiety Inventory

## Footnotes

1 “Adequate knowledge” was defined as > 50% correct on objective knowledge of treatment items (i.e., at least 20 out of 38 marks). To have adequate knowledge, participants must either get correct: i) all conceptual knowledge items (18 marks) *plus* at least 2 marks on numerical knowledge items (2 marks); or ii) all numerical knowledge items (20 marks).
